# GWAS-Top Polymorphisms Associated With Late-Onset Alzheimer Disease in Brazil: Pointing Out Possible New Culprits Among Non-Coding RNAs

**DOI:** 10.3389/fmolb.2021.632314

**Published:** 2021-07-05

**Authors:** Gabriela Canalli Kretzschmar, Nina Moura Alencar, Saritha Suellen Lopes da Silva, Carla Daniela Sulzbach, Caroline Grisbach Meissner, Maria Luiza Petzl-Erler, Ricardo Lehtonen R. Souza, Angelica Beate Winter Boldt

**Affiliations:** ^1^ Laboratory of Human Molecular Genetics, Postgraduate Program in Genetics, Department of Genetics, Federal University of Paraná, Curitiba, Brazil; ^2^ Laboratory of Polymorphism and Linkage, Postgraduate Program in Genetics, Department of Genetics, Federal University of Paraná, Curitiba, Brazil

**Keywords:** Alzheimer’s disease, GWAS, APOE, BIN1, CELF1, lncRNA, miRNA

## Abstract

Several genome-wide association studies (GWAS) have been carried out with late-onset Alzheimer’s disease (LOAD), mainly in European and Asian populations. Different polymorphisms were associated, but several of them without a functional explanation. GWAS are fundamental for identifying loci associated with diseases, although they often do not point to causal polymorphisms. In this sense, functional investigations are a fundamental tool for discovering causality, although the failure of this validation does not necessarily indicate a non-causality. Furthermore, the allele frequency of associated genetic variants may vary widely between populations, requiring replication of these associations in other ethnicities. In this sense, our study sought to replicate in 150 AD patients and 114 elderly controls from the South Brazilian population 18 single-nucleotide polymorphisms (SNPs) associated with AD in European GWAS, with further functional investigation using bioinformatic tools for the associated SNPs. Of the 18 SNPs investigated, only four were associated in our population: rs769449 (*APOE*), rs10838725 (*CELF1*), rs6733839, and rs744373 (*BIN1–CYP27C1*). We identified 54 variants in linkage disequilibrium (LD) with the associated SNPs, most of which act as expression or splicing quantitative trait loci (eQTLs/sQTLs) in genes previously associated with AD or with a possible functional role in the disease, such as *CELF1*, *MADD*, *MYBPC3*, *NR1H3*, *NUP160*, *SPI1*, and *TOMM40*. Interestingly, eight of these variants are located within long non-coding RNA (lncRNA) genes that have not been previously investigated regarding AD. Some of these polymorphisms can result in changes in these lncRNAs’ secondary structures, leading to either loss or gain of microRNA (miRNA)-binding sites, deregulating downstream pathways. Our pioneering work not only replicated LOAD association with polymorphisms not yet associated in the Brazilian population but also identified six possible lncRNAs that may interfere in LOAD development. The results lead us to emphasize the importance of functional exploration of associations found in large-scale association studies in different populations to base personalized and inclusive medicine in the future.

## Introduction

Late-onset Alzheimer’s disease (LOAD) is a neurodegenerative disease responsible for most dementia cases worldwide in the elderly population (Lane et al., 2018). The neuropathological features are the accumulation of β-amyloid (Aβ) plaques and neurofibrillary tangles (NFTs), leading to neuronal death and cerebral atrophy (Braak and Braak, 1991). LOAD is a complex disease, with the influence of several genetic factors, in addition to environmental factors. Although there are numerous studies on LOAD with the most diverse approaches, the mechanisms involved in this disease remain poorly understood.

Genome-wide association studies (GWAS) started in 2006 and have grown exponentially in number (Buniello et al., 2019), allowing for the identification of countless genetic variants associated with diseases and phenotypic traits. However, most of them are not directly implicated in the phenotype, being markers in high linkage disequilibrium (LD) with the unknown regulatory or structural causal variant (Maurano et al., 2012; Zhu et al., 2016). Besides, most GWAS were performed in European and Asian populations, hindering the extrapolation of results for populations of other or mixed ancestries, given the possible difference of gene or haplotype frequencies (Nédélec et al., 2016). Currently, over 90 GWAS have been performed with LOAD (GWAS Catalog). They led to the identification of different polymorphisms, mostly in intronic or intergenic regions, possibly modulating the susceptibility to disease in the populations where they were analyzed, but most were not investigated nor replicated yet in Latin American populations (Kretzschmar et al., 2020).

In this context, our study sought to replicate in the South Brazilian population some of the single-nucleotide polymorphism (SNP) alleles found associated with LOAD in GWAS performed in European-derived populations, as well as to evaluate possible functional explanations for these associations.

## Materials and Methods

**FIGURE 1:**
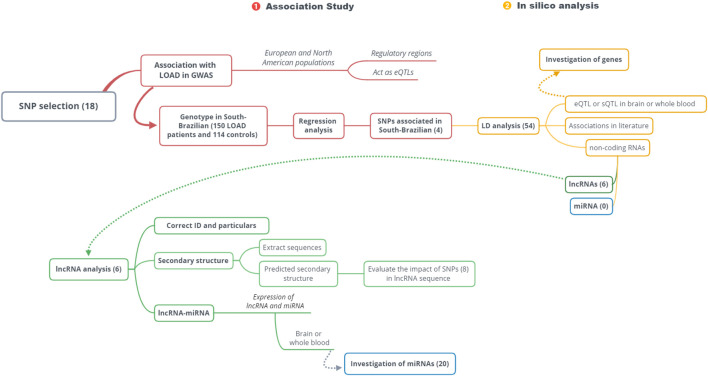
Workflow. This study is divided into two stages: 1, association study; 2, *in silico* analysis. Only the associated variants in the studied population (South Brazilian) followed *in silico* analysis. SNP: single-nucleotide polymorphism; LOAD: late-onset Alzheimer’s disease; GWAS: genome-wide association studies; eQTL: expression quantitative trait locus; sQTL: splicing quantitative trait locus; LD: linkage disequilibrium; lncRNAs: long non-coding RNAs; miRNAs: microRNAs.

To understand this work’s multistep nature, we represent it as a two-stage workflow (Figure 1), showing first, the replication in patients from South Brazil of European-associated LOAD variants, followed by the *in silico* investigation of replicated associations.

### Ethical Approval

This study was performed in accordance with the ethical standards of the Research Ethics Committee of the Health Sciences Sector (Federal University of Paraná) (CAAE: 55965316.1.0000.0102), according to Resolution 466/2012 of the National Health Council and the 1964 Helsinki Declaration and its later amendments or comparable ethical standards. Informed consent was obtained from all participants included in the study.

### Association Study

#### Research Participants

The 150 LOAD patients were recruited from the Clinical Hospital of the Federal University of Paraná (HC-UFPR) (*n* = 97) and the Institute of Neurology of Paraná (INC) (*n* = 53). To be included, patients should be diagnosed with LOAD based on the clinical history and cognitive tests (Frota et al., 2011). Forms of dementia other than LOAD, inconclusive diagnosis, and less than 60 years of age were exclusion criteria. The elderly controls (114 individuals) were confirmed to be healthy and neurologically normal, according to their medical history and scores in the Mini–Mental State Examination (MMSE) scale. Individuals with infectious diseases or of 60–65 years of age and family history of AD were excluded from both groups. Since *APOE* (*apolipoprotein E*) rs7412 and rs429358 polymorphisms are strongly associated with LOAD (Lane et al., 2018), we previously genotyped the samples by TaqMan real-time PCR (Life Technologies 4351379) to correct for the presence of the LOAD-associated alleles in logistic regression analyses. Further sample descriptions can be found in Table 1.

**TABLE 1:** Demographic and clinical characteristics of research participants.

Variable	Controls, *n* = 114 (%)	Patients, *n* = 150 (%)
Male (%)	28 (24.8)	52 (34.7)
Average age (min–max)	70.8 (60–99)	75.6 (60–90)
*APOE ε4+* (%)	20 (17.7)	70 (47.9)
**Predominant ethnicity**		
Euro-Brazilian (%)	92 (80.7)	120 (80)
Admixed (%)	20 (17.5)	28 (18.7)
Indeterminate (%)	2 (1.8)	2 (1.3)

Ancestry was self-reported. The proportions agree with the South Brazilian population’s actual genomic composition (Lima-Costa et al., 2015). We emphasize that Euro-Brazilian participants are descendants of Europeans but are admixed. APOE: apolipoprotein E.

### Polymorphism Selection and Genotyping

Total DNA was extracted from peripheral blood using the salting-out standard protocol (Lahiri and Numberger, 1991). We genotyped eighteen single-nucleotide polymorphisms (SNPs), listed in Table 2. SNP selection was based on their association with LOAD in GWAS conducted with European and North American populations with European ancestry, accessing the GWAS Catalog (Buniello et al., 2019). These populations are more similar to the South Brazilian population, whose ancestry has a strong European component, mainly from the Iberian and Tuscany regions (Lima-Costa et al., 2015). Selected SNPs should also be located in regulatory regions or act as expression quantitative trait loci (eQTLs). SNP rs3857059 was the only exception, since it was originally associated with Parkinson’s disease in a GWAS. We selected it to evaluate if it could be associated with LOAD in our population.

**TABLE 2:** Single-nucleotide polymorphisms (SNPs) selected for this study and their minor allele frequencies.

Region/closest gene	SNP	Alleles (maj./min.)	CHR	Position GRCh38.p12	Region	Control		Patients		GWAS population* (%)				AD	References
						%	N^&^	%	N^&^	CEU	TSI	IBS	YRI		
*ABCA7*	rs4147929	G/A	19	1063444	Intron	17.7	226	17.0	300	18	22	23	0	A	Lambert et al. (2013)
*ADAMTS9*	rs704454	T/C	3	64941350	Intron	24.7	218	25.3	288	26	30	29	19	?	Kamboh et al. (2012)
*APOE*	rs769449	G/A	19	44906745	Intron	7.14	224	20.6	296	15	9	8	0	A	Cruchaga et al. (2013)
*BIN1–CYP27C1*	rs6733839	C/T	2	127135234	Intergenic	33.04	224	40.0	300	40	42	31	39	T	Lambert et al. (2013)
*BIN1–CYP27C1*	rs744373	A/G	2	127137039	Intergenic	28.3	222	34.8	290	30	29	25	56	?	Hollingworth et al. (2011)
*CD2AP*	rs10948363	A/G	6	47520026	Intron	25.6	226	26.3	300	28	24	24	10	G	Lambert et al. (2013)
*CD33*	rs3865444	C/A	19	51224706	Promoter	32.6	224	33.4	296	32	27	29	1	?	Lambert et al. (2013)
*CELF1*	rs10838725	T/C	11	47536319	Intron	30.7	228	28.7	300	34	34	29	0	C	Lambert et al. (2013)
*CLU*	rs11136000	C/T	8	27607002	Intron	41.6	226	40.7	300	37	40	36	60	C	Harold et al. (2009), Lambert et al. (2009)
*CTNNA2*	rs2974151	C/G	2	79926171	Intron	14.6	226	12.2	296	12	15	11	15	?	Cummings et al. (2012)
*EPHA1*	rs11771145	G/A	7	143413669	Intron	36.7	226	32.7	300	35	36	35	59	?	Lambert et al. (2013)
*INPP5D*	rs35349669	C/T	2	233159830	Intron	38.5	226	36.3	300	42	43	40	5	T	
*MS4A6A*	rs610932	G/T	11	60171834	Intergenic	41.5	224	41.2	296	52	53	43	42	?	Hollingworth et al. (2011)
*PICALM*	rs3851179	C/T	11	86157598	Intergenic	33.6	226	33.4	296	43	36	35	5	C	Harold et al. (2009)
*ZCWPW1*	rs1476679	T/C	7	100406823	Intron	20.8	226	23.0	300	32	31	21	0	?	Lambert et al. (2013)
*PTK2B*	rs28834970	T/C	8	27337604	Intron	35.0	226	35.3	300	34	33	35	18	C	Lambert et al. (2013)
*SLC24A4*	rs10498633	G/T	14	92460608	Intron	17.3	226	18.0	300	18	16	22	11	?	Lambert et al. (2013)
*SNCA* ^***A***^	rs3857059	A/G	4	89754087	Intron	19.9	226	17.1	292	8	7	7	65	?	Simón-Sánchez et al. (2009), Linnertz et al. (2014)

**A**: although this SNP does not appear associated with AD GWAS, it is commonly associated with Parkinson’s disease (PD) GWAS. We selected this SNP to see if we would find any association with LOAD in our population. Allele frequencies of the investigated SNPs: three European populations and one African population are presented for comparative purposes. SNPs: single-nucleotide polymorphism; ?: no information or both alleles were AD-associated in independent studies; maj.: major allele; min.: minor allele; CEU: population of Utah with northern and western European ancestry; TSI: population of Toscana, Italy; IBS: Iberian population, Spain; YRI: African population of Yoruba. ^*^Allele frequencies according to data from the 1000 Genomes Project (Consortium, 2010); ABCA7: *ATP-binding cassette subfamily A member 7*; ADAMTS9-AS2: ADAMTS9 *antisense RNA 2*; APOE: *apolipoprotein E*; BIN1: *bridging integrator 1*; CYP27C1: *cytochrome P450 family 27 subfamily C member 1*; CD2AP: *CD2-associated protein*; CD33: *CD33 molecule*; CELF1: *CUGBP Elav-like family member 1*; CLU: *clusterin*; CTNNA2: *catenin alpha 2*; EPHA1: *EPH receptor A1*; INPP5D: *inositol polyphosphate-5-phosphatase D*; MS4A6A: *membrane-spanning 4-domains A6A*; PICALM: *phosphatidylinositol-binding clathrin assembly protein*; ZCWPW1: *zinc finger CW-type and PWWP domain containing 1*; PTK2B: *protein tyrosine kinase 2 beta*; *SLC24A4*: *solute carrier family 24 member 4*; SNCA: *synuclein alpha*; & = number of chromosomes. Some samples were excluded due to low genotyping quality. The maximum sample number was 150 patients (300 chromosomes) and 114 elderly controls (228 chromosomes).

Genotyping was performed by mass spectrometry using the iPLEX MassARRAY Platform (Sequenom, San Diego, CA) at Auckland University (NZ).

### Association Analysis

Allelic and genotypic frequencies were obtained by direct counting, and their distribution was evaluated according to the Hardy–Weinberg equilibrium hypothesis (applied by PLINK v.1). Differences between the distributions of polymorphisms in patients and controls were compared using the exact Fisher test and binary multivariate logistic regression (dominant, recessive, and additive models) with STATA v.9.2 (Statacorps, Lakeway Drive, TX), correcting for independent variables (Table 3). Only independent variables with *p*-values lower than 0.22 were considered for multivariate regression analysis. Only models with intercept values corresponding to *p* ≤ 0.05 were considered. The *p*-values were corrected for multiple testing using the false discovery rate (FDR) method (Benjamini and Hochberg, 1995), performed in R language 3.6.1, through the Stats package (R Development Core Team, 2011). Corrected *p*-values (Pc) lower than 0.05 were considered significant.

**TABLE 3:** Results of univariate analysis for all available variables.

Independent variable	OR	95% CI	*p*-Value
Ethnicity	0.93	0.49–1.76	0.827
Sex	0.62	0.36–1.07	**0.086**
Schooling	0.88	0.73–1.07	**0.215**
Smoking habit	2.28	1.27–4.09	**0.006**
Alcoholism	3.24	1.45–7.22	**0.004**
Diabetes	0.81	0.45–1.45	0.476
Cholesterol	0.75	0.44–1.29	0.299
Hypertension	0.71	0.40–1.25	0.232
BMI	0.65	0.45–0.96	**0.029**
AD in family	4.87	2.25–10.53	**<0.000**
*e2*	1.85	0.73–4.66	**0.192**
*e3*	0.13	0.03–0.57	**0.007**
*e4*	4.28	2.39–7.66	**<0.000**

Variables with *p*-values lower than 0.220 were considered for multivariate regression analysis (in bold); BMI: body mass index.

### In Silico Analysis

To further explore the genetic associations found in the South Brazilian population, we performed *in silico* analysis using publicly available tools and databases (cited in Supplementary Table S1).

#### Linkage Disequilibrium

Since many of the variants found associated in a GWAS are not responsible for the disease and probably act as tag SNPs, we performed the LD analysis for the variants that remained associated after correction for independent variables and FDR. We used LDlink, a web-based tool that uses the publicly available reference haplotypes from Phase 3 of the 1000 Genomes Project to calculate population-specific measures of LD. Using the proxy tool of LDlink, we considered as LD variants only those with r2 > 0.8 in at least one of the following populations: from Utah with northern and western European ancestry (CEU), from Tuscany (TSI), and from the Iberian Peninsula (IBS).

#### Search for Associations in the Literature

For all variants considered in LD and the SNPs associated in this study, we searched for previous associations with AD in the literature through PubMed, Web of Science, and Google Scholar databases. As search terms, we initially used only each variant to check all the associations already reported. Later, we filtered for articles that included the word “Alzheimer” in the title or abstract.

#### Expression and Splicing Quantitative Trait Loci (eQTL/sQTL)

For SNPs associated in this study and all variants in LD with them, we evaluated their possible role as eQTL or sQTL in brain tissue and/or whole blood (GTEx and Braineac). All genes whose expression level was associated with these eQTLs and sQTLs, as well as the genes where the variants in LD were located, were investigated for their characteristics, expression, regulation, structure, function of the encoded protein, protein network, and possible associations with diseases reported in the literature.

#### Investigation of Non-Coding RNAs Possibly Related to AD Through the Associated or Linkage Disequilibrium Variants

For all the SNPs associated in this study and their respective variants in LD, we evaluated the physical location, especially if they occur within the sequence of non-coding RNA (ncRNA) genes, where they may affect the structure and function of the ncRNA. For variants in long non-coding RNAs (lncRNAs), we analyzed whether they could lead to a change in their secondary structure or a gain/loss of microRNA (miRNA)-binding sites, disturbing lncRNA–miRNA interactions (since both can be endogenous competitors). We also searched for information on expression, function, and previous associations of these lncRNAs in the literature and corresponding databases. All lncRNAs were mapped according to their genomic coordinates using UCSC (GRch38. p13). We also looked for genes within 2 kb distance from the 5′ and 3′ sequence limits of these lncRNA genes.

#### Secondary Structure of Long Non-Coding RNA Prediction

All variants found in the sequence of lncRNA genes occurred within exons, possibly resulting in a structural and, consequently, a functional change. The secondary structure of the lncRNAs was predicted through the online version of the RNAfold web server, based on the Vienna RNA package. We obtained the lncRNA sequences through NONCODE and searched for the variant’s location within the lncRNA using the Ensembl and UCSC databases (GRch38. p13). For each lncRNA, we generated secondary structures for both alleles of the variant, using the calculation of minimum free energy (MFE) and positional entropy (the input sequences are available at https://osf.io/njau3/?view_only=949c6b1c91c94e33b2c3e4d152f82a0e). To assess the mutation’s possible impact on the structure, we considered the conformational change of the molecule and the *p*-value provided by the lncRNASNP2 database. This *p*-value is empirical, generated by the SNP’s position, the GC content of the molecule, and the size of the sequence (*p* < 0.2 indicating possibly harmful).

#### Investigation of MicroRNAs That Are Possibly Affected by the Presence of Variants in Long Non-Coding RNAs

For the miRNAs affected by the variants identified in the lncRNA gene sequences, we investigated their tissue expression, lncRNA–miRNA interaction, pathway enrichment, and genes regulated by them, as well as previous associations in the literature. For the analysis of pathways, we considered only those related to Alzheimer’s disease pathophysiology through miRPath (KEGG and GO pathways).

## Results

### Association Study

Genotype distributions were in Hardy–Weinberg equilibrium for both patients and controls (Table 2, Table 4, and Supplementary Table S2). Using binary logistic regression, we selected nine independent variables with a tendency or an association with LOAD (*p*-value < 0.220). We used them in multiple regression models to correct any associations with polymorphisms (Table 3, Table 4, and Supplementary Table S2). Of the 18 selected SNPs, only four remained associated after correction for independent variables and FDR: rs769449 (*APOE*); rs6733839 and rs744373 (*BIN1–CYP27C1*); and rs10838725 (*CELF1*) (Table 4).

**TABLE 4:** Significant results of the polymorphisms investigated with LOAD.

Region	SNP		OR	95% CI	P	Pc#	IV	HWE	
								CON	PAT
*APOE*	rs769449	A/A*	–	–	–			1	1
		A/G	0.84	0.32–2.25	0.736				
		G/G	**0.28**	**0.14**–**0.57**	**<0.000**	**0.0002**	**BMI**		
		A+	**3.55**	**1.76**–**7.19**	**<0.000**	**0.0002**	**BMI**		
		G+*	–	–	–				
		Additive model	**3.49**	**1.80**–**6.76**	**<0.000**	**0.0002**	**BMI**		
*BIN1–CYP27C1*	rs6733839	C/C	0.55	0.29–1.05	0.070		BMI, e4	0.281	0.865
		C/T	1.30	0.70–2.41	0.406				
		T/T	1.96	0.76–5.05	0.165				
		C+	0.51	0.20–1.32	0.165				
		T+	1.81	0.95–3.43	0.070		BMI, e4		
		Additive model	**1.62**	**1.01**–**2.59**	**0.045**	**0.049**	**BMI, e4**		
*BIN1–CYP27C1*	rs744373	A/A	0.69	0.36–1.29	0.246			0.242	0.856
		A/G	0.98	0.51–1.86	0.948				
		G/G	**2.83**	**1.04**–**7.66**	**0.041**	**0.049**	**e4**		
		A+	0.32	0.10–1.0	0.051		BMI, e4		
		G+	1.45	0.77–2.74	0.246				
		Additive model	1.55	0.96–2.51	0.076		BMI, e4		
*CELF1*	rs10838725	C/C	**0.21**	**0.04**–**0.99**	**0.049**	**0.049**	**BMI, e4**	0.660	0.110
		C/T	1.48	0.80–2.75	0.212				
		T/T	0.95	0.52–1.75	0.875				
		C+	1.05	0.57–1.93	0.875				
		T+	**4.88**	**1.01**–**23.60**	**0.049**	**0.049**	**BMI, e4**		
		Additive model	0.83	0.50–1.36	0.453				

The values are the result of logistic regression performed by STATA. Bold: significant *p*-value; underline: trend; OR: *odds ratio*; CI: confidence interval; P= *p*-value; Pc#: *p*-value corrected for false discovery rate; IV: independent variable; HWE: Hardy–Weinberg equilibrium; PAT: patients; CON: controls; BMI: body mass index; +: allele carrier; *: it is not possible to calculate since all the controls have the rs769449*G allele. APOE: *apolipoprotein E*; BIN1: *bridging integrator 1*; CYP27C1: *cytochrome P450 family 27 subfamily C member 1*; CELF1: *CUGBP Elav-like family member 1*. All results are in Supplementary Table S2.

### In Silico Analysis

#### Identification of Variants in Linkage Disequilibrium, Expression and Splicing Quantitative Trait Locus Effect, and Interference in Non-Coding RNAs

We investigated variants in LD with the four associated SNPs in the CEU, TSI, and IBS populations and found a total of 54 SNPs in LD with them (r2 > 0.8, Supplementary Table S3). For rs10838725 (*CELF1*), we found 49 variants in LD, present in the following genes: *CELF1*, *MTCH2*, *AGBL2*, *FNBP4*, and *NUP160*, and in intergenic regions: *NDUFS3*–*FAM180B*, *C1QTNF4*–*MTCH2*, and *NUP160*—*PTPRJ* (Supplementary Figure S1). In addition to rs10838725 (*CELF1*) itself, of the 49 variants in LD with this SNP, 40 act as eQTLs for *NDUFS3*, *FAM180B*, *SLC39A13*, *C1QTNF4*, *MYBPC3*, *PTPRJ*, *FNBP4*, *MADD*, *ARHGAP1*, *ARFGAP2*, *PTPMT1*, and *ACP2* and sQTLs for *SLC39A13* and *SPI1* in brain regions and/or whole blood. Two variants in LD with rs10838725 (rs71457224 and rs10769282) occur within a lncRNA (*NONHSAT021264.2*) gene, where probably rs10769282 results in the loss of a binding site for hsa-miR-373-5p.

There are three SNPs in LD with rs769449 (*APOE*), one of which (rs429358) is responsible for the APOE*ε4 allele. rs7256200 and rs10414043 act as eQTLs for *RSPH6A* in intralobular white matter (Supplementary Figure S2). Besides that, rs769449 itself, rs10414043, and rs429358 can lead to alternative splicing (sQTL) of the *TOMM40* pre-mRNA in the cerebellar hemisphere and cerebellum. In addition, rs429358 is also an sQTL for the *APOE* gene in the basal ganglia. rs10414043 and rs7256200 occur within the *NONHSAT179794.1* lncRNA gene. According to *in silico* prediction, rs10414043 leads to a gain (hsa-miR-5089-3p) and a loss (hsa-miR-1273g-3p, hsa-miR-4252, and hsa-miR-1227-3p) of miRNA-binding sites in this lncRNA, whereas rs7256200 leads to a loss of the hsa-miR-4284–binding site. Furthermore, rs429358 is located within two lncRNA genes (*NONHSAT066732.2* and *NONHSAT179793.1*), resulting in a gain (hsa-miR-4479) and a loss (hsa-miR-4479) of miRNA-binding sites in *NONHSAT066732.2* and a gain (hsa-miR-6869-3p) in *NONHSAT179793.1*.

rs6733839 (*BIN1–CYP27C1*) is in LD only with rs4663105. This SNP acts as an eQTL for the *BIN1* gene in the cerebellum and whole blood. This SNP also occurs within a lncRNA gene (*NONHSAT187478.1*), resulting in a gain (hsa-miR-6776-5p and hsa-miR-4455) and a loss (hsa-miR-6839-3p) of miRNA sites. Another SNP investigated in the *BIN1*–*CYP27C*1 region (rs744373) is in LD only with rs730482. None of them act like an eQTL/sQTL, but both occur within the *NONHSAT182593.1* lncRNA gene, where rs744373 can lead to a gain (hsa-miR-5008-5p) or loss (hsa-miR-2467-5p, hsa-miR-657, and hsa-miR-6822-3p) of miRNA-binding sites and rs730482 can result in the loss of various miRNA-binding sites (hsa-miR-192-5p, hsa-miR-215-5p, hsa-miR-4766-3p, and hsa-miR-1224-3p).

#### Characterization and Secondary Structure of Candidate Long Non-Coding RNAs

We found six lncRNAs potentially involved in LOAD, carrying eight investigated variants, of which only rs744373 was associated in the South Brazilian population (Table 5). There is a general lack of information about these lncRNAs, mostly derived from databases. Except for *NONHSAT066732.2*, for which it is unknown whether it is not expressed in this tissue or has just not been analyzed, all others are expressed in the brain.

**TABLE 5:** Characterization of lncRNAs potentially involved in AD.

lncRNA ID	Other IDs	Position (GRCh38)	Genes within 2 Kb	Class.	BP	Variant	miRNA target		p*
							Gain	Loss	
*NONHSAT021264.2*	*lnc-FAM180B-2:1*	chr11:47602805-47611134	-	lincRNA	1,280	rs71457224	-	-	-
						rs10769282	-	hsa-miR-373-5p	0.6488
*NONHSAT179794.1*	*AC011481.3*	chr19:44909374-44914968	*APOE*	?	5,594	rs10414043	hsa-miR-5089-3p	hsa-miR-1273g-3p	0.3916
								hsa-miR-4252	
			*APOC1*					hsa-miR-1227-3p	
						rs7256200	-	hsa-miR-4284	0.2019
*NONHSAT066732.2*	*lnc-ZNF296-6:1*	chr19:44907906-44909013	*APOE*; *AC011481.3*	Antisense	526	rs429358	hsa-miR-4479	hsa-miR-147b	0.0666
*NONHSAT179793.1*	-	chr19:44907758-44909389	*APOE*; *AC011481.3*; *lnc-ZNF296-6:1*	?	1,051	rs429358	hsa-miR-6869-3p	-	0.0666
*NONHSAT187478.1*	HSALNT0039381	chr2:127133598-127135107	-	lincRNA	1,509	rs4663105	hsa-miR-6776-5p	hsa-miR-6839-3p	0.9863
							hsa-miR-4455		
*NONHSAT182593.1*	-	chr2:127116083-127139365	*lnc-TEX51-4*	lincRNA	4,459	rs744373	hsa-miR-5008-5p	hsa-miR-2467-5p	0.9571
								hsa-miR-657	
								hsa-miR-6822-3p	
						rs730482	-	hsa-miR-192-5p	0.4906
								hsa-miR-215-5p	
								hsa-miR-4766-3p	
								hsa-miR-1224-3p	

Class.: lncRNA classification; bp: base pairs; * *p*-value of the possibility of SNP impacting the lncRNA structure [this *p*-value is empirical, being generated in silico, through the position of the SNP, the GC content of the molecule, and the size of the sequence (*p* < 0.2 = possibly harmful)].

**FIGURE 2:**
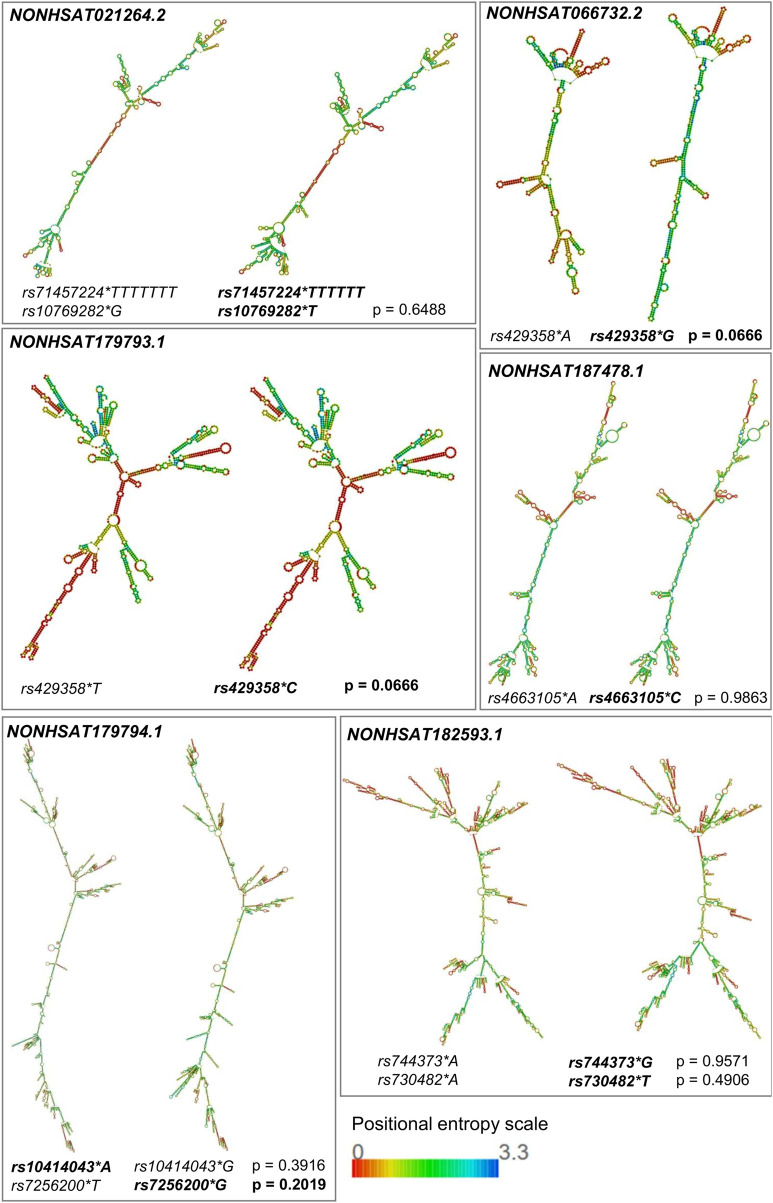
Prediction of secondary structures of lncRNAs possibly involved in AD. The secondary structures of the lncRNAs possibly involved in AD were predicted by the RNAfold web server based on the Vienna RNA package, considering the calculation of minimum free energy (MFE) and positional entropy. Structures were generated for both alleles (alleles in bold are deemed to have a possible harmful role). The impact of these mutations on the structures was established by visually changing the molecule and the *p*-value provided by lncRNASNP2. This *p*-value is empirical, being generated *in silico*, through the SNP’s position, the GC content of the molecule, and the size of the sequence (*p* < 0.2 = possibly harmful). Within the positional entropy scale, low entropy (red) leads to little structural flexibility, making the prediction more reliable, while regions of high entropy (violet) may have several alternative structures.

All the variants occur within the mature lncRNA sequence. According to results from the lncRNASNP2 database, which provides an empirical *p*-value for structural lncRNA damage (*p* < 0.2 = possibly harmful), we predicted a possible harmful shift in the secondary structure of *NONHSAT179794.1* (due to rs7256200, *p* = 0.2019), *NONHSAT066732.2*, and *NONHSAT179793.1* (due to rs429358, *p* = 0.0666 for both). Besides, a structural variation is clearly noticeable for *NONHSAT021264.2* (rs71457224 and rs10769282) and *NONHSAT182593.1* (rs744373 and rs730482) but possibly is not harmful (Figure 2).

Through ncRPheno, a comprehensive database that provides experimentally supported associations between non-coding RNAs and disease phenotypes, we found that *NONHSAT179794.1*, *NONHSAT179793.1*, *NONHSAT187478.1*, and *NONHSAT182593.1* could lead to neurodegenerative disorders (including LOAD) (Supplementary Figure S3).

#### MicroRNA-Binding Sites Affected by Single-Nucleotide Polymorphisms in Long Non-Coding RNAs

The presence of SNPs in lncRNAs could create or disrupt a miRNA-binding site. We identified 20 miRNAs whose binding sites were affected by SNPs located within lncRNAs, of which seven gained and thirteen lost a binding site (Table 5).

Of the 20 miRNAs, 14 are known to be expressed in the brain. All others do not have enough data to determine whether they are expressed or not in the brain. Only four were previously associated with any neurological disease or with AD risk factors: hsa-miR-373-5p with schizophrenia (Pala and Denkçeken, 2020); hsa-miR-657 with type 2 diabetes (Lv et al., 2008); hsa-miR-192-5p with venous thrombosis and type 2 diabetes (Lu et al., 2020; Rodriguez-Rius et al., 2020); and has-miR-147b with negative regulation of the inflammatory response (van Scheppingen et al., 2018).

## Discussion

GWAS are extremely relevant for identifying genome regions associated with a disease. However, most of the associated loci occur in non-coding areas, turning it difficult to establish causality with the disease (Albert and Kruglyak, 2015). Often, the associations reflect the deregulation of gene expression, resulting from changes caused by the presence of variants in regulatory regions (promoters, enhancers) or ncRNA genes, or even from LD with variants that act as eQTLs/sQTLs (Albert and Kruglyak, 2015; Hu et al., 2019). Also, the associations found are closely related to the genetic background of the assessed population. Most of the GWAS carried out with LOAD used samples from European populations or with European ancestry (GWAS Catalog), which do not necessarily reflect the genetic diversity of other populations. Thus, we sought to replicate in the South Brazilian sample some of the main associations reported in LOAD-GWAS performed with European-derived populations, investigating the possible functional role of these variants in LOAD development.

The *APOE* (*apolipoprotein E*) gene presents three distinct allelic variants (*ε2*, *ε3*, and *ε4*). Their product is probably involved in Aβ production and/or clearance, neuroinflammation, synaptic loss, and tau hyperphosphorylation, important for the development and progression of LOAD (Yu et al., 2014; Yamazaki et al., 2019). The rs429358*C allele corresponds to the ε4 isoform and is considered the most critical genetic susceptibility factor for LOAD development (Corder et al., 2008; Castellano et al., 2011; Lambert et al., 2013). This SNP allele is in LD with rs769449*A, associated in our sample with susceptibility to LOAD. rs429358 is located within two lncRNAs genes (*NONHSAT179793.1* and *NONHSAT066732.2*), leading to a change in the secondary structure of *NONHSAT066732.2*, resulting in the loss of the hsa-miR-147b–binding site and the gain of the hsa-miR-4479–binding site. Increased expression of hsa-miR-147b is associated with down-regulation of the inflammation driven by activated astrocytes (van Scheppingen et al., 2018). Due to the loss of miRNA–lncRNA interaction caused by rs429358*G, there is possibly a greater availability of hsa-miRNA-147b, reducing the inflammatory response. While this may seem beneficial, it possibly harms the clearance of Aβ plaques promoted by inflammatory elements. Also, through pathway enrichment analysis, we observed that hsa-miR-4479 is involved in the GABAergic pathway regulating the expression of *CACNA1A*, *SLC32A1*, *PRKX*, and *SLC12A5* genes (miRPath). With the addition of a binding site in *NONHSAT066732.2*, this miRNA may be sequestered, leading to an imbalance in GABAergic signaling, which has been considered to be involved in LOAD pathology (Li et al., 2016). Besides, rs429358 is located in a CpG island and may impact DNA methylation. Foraker et al. (2015) demonstrated a difference in this region’s methylation profile between individuals with AD and controls. rs769449 is also in LD with two other variants (rs10414043 and rs7256200) located in the lncRNA *NONHSAT179794.1*, with rs7256200 leading to a structural change in the lncRNA molecule. Recent studies have shown that lncRNAs can affect expression of genes found in the proximity (within 2 Kb) (Engreitz et al., 2016). Thus, both *NONHSAT179793.1* and *NONHSAT066732.2* may interfere with *APOE* regulation. However, the association observed in our study with rs769449 is possibly related to its LD with other variants having high regulatory potential. Other studies have already shown that the change of *G* for an *A* allele, creating the rs769449*A allele, may favor an open chromatin state for the *APOE* gene, along with a correspondent strong H3K4Me3 signal (trimethylation of lysine 4 in histone H3) (Ryu et al., 2016; Babenko et al., 2018). Furthermore, the rs769449*A allele is absent in older people with greater longevity, being related to poor LOAD prognoses, such as inferior recovery of late verbal memory and faster cognitive decline (Soerensen et al., 2013; Zhang and Pierce, 2014; Arpawong et al., 2017).

Variants of *BIN1* (*bridging integrator 1*) commonly show the second highest odds ratios for LOAD, lagging only behind *APOE* variants (Tan et al., 2013; Crotti et al., 2019; Franzmeier et al., 2019). It is involved in endocytosis, sustained cytoskeleton integrity, regulation of the tau peptide, and probably inflammation, calcium homeostasis, and apoptosis (Tan et al., 2013; Crotti et al., 2019; Franzmeier et al., 2019; Sartori et al., 2019; Thomas et al., 2019). Tau is a microtubule-associated protein which, under a pathological condition, is phosphorylated (pTau) and assembles into insoluble aggregates (neurofibrillary tangles), leading to synaptic dysfunction and neural cell death, which plays an essential role in the development and progression of LOAD (Franzmeier et al., 2019; Thomas et al., 2019). Our study validated the association of the two rs6733839 and rs744373 SNPs, located in the *BIN1–CYP27C1* region. rs6733839 carriage is associated with higher pTau181 levels in CSF (Crotti et al., 2019). Homozygote individuals for rs6733839*T show worse episodic memory (Greenbaum et al., 2016). This SNP is in LD with rs4663105, which occurs in the *NONHSAT187478.1* lncRNA gene, possibly associated with neurodegenerative diseases (ncRPheno). Also, rs4663105 is an eQTL for *BIN1* in the cerebellum and whole blood (GTEx). rs744373*G was recently associated with LOAD in the Colombian population (Moreno et al., 2017) and was found to increase tau pathology in LOAD (Franzmeier et al., 2019). We found this SNP and rs730482 (both in LD) in *NONHSAT182593.1* lncRNA, possibly associated with neurodegenerative diseases (ncRPheno).

The *CELF1* (*CUGBP Elav-like family member 1*) gene encodes CELF1 protein, a RNA-binding protein related to the regulation of different post-transcriptional events and alternative splicing, mRNA translation, and mRNA stability (Beisang et al., 2012; Bateman et al., 2017). During alternative RNA processing, the protein can select the splicing target site by binding to U/G-rich elements in the transcript sequence, leading to mRNA decay and controlling translation efficiency (preprint David et al., 2020). The rs10838725 polymorphism has been associated with LOAD in a GWAS (Lambert et al., 2013; Karch et al., 2016; Marioni et al., 2018). However, this SNP possibly is not the only causal SNP, since it occurs in LD with several variants distributed in distinct genes, intergenic regions, and *NONHSAT021264.*2 lncRNA region (rs71457224 and rs10769282). Besides that, in this LD block, several variants act as eQTLs/sQTLs in the brain and whole blood, altering the expression of many genes already related to LOAD or other neurological diseases in human or animal studies, such as *CELF1* itself, *MADD*, *MYBPC3*, *NR1H3*, *NUP160*, *SPI1*, and *TOMM40* (Natunen et al., 2013; Karch et al., 2016; Dourlen et al., 2017; Huang et al., 2017; Katsumata et al., 2019; Zhu et al., 2019, 2020; Lutz et al., 2020).

Our work has some limitations: it does not share the statistical power of GWAS for validation of allelic associations, and we might have missed some true associations with alleles of lower frequency due to the smaller sample size. Furthermore, the secondary structures of lncRNAs are the result of an *in silico* analysis. This structural prediction does not consider the huge complexity of possible interactions within a RNA molecule and its interactions with other molecules, which can dramatically alter its structure. The *in silico* analysis results from the compilation of information obtained in online databases, some of which lack experimental validation.

Nonetheless, we replicated in the South Brazilian population the associations already reported with LOAD in a European GWAS for *APOE*, *BIN1*, and *CELF1*. Of the eighteen polymorphisms analyzed, only four remained associated with the South Brazilian population (these are the first confirmatory results for these polymorphisms in the Brazilian population), corroborating previous studies of our group (Kretzschmar et al., 2020). The low replication rate in South Brazilians is due to the admixture with other human groups, such as Amerindians and Africans, which causes South Brazilians to differ from Europeans, despite their major Iberian and Tuscany origin (Braun-Prado et al., 2000; Probst et al., 2000; Pena et al., 2020). This highlights the importance of replication of associated variants in different ethnicities, to contribute to a more personalized and inclusive medicine.

Furthermore, the need for functional exploration of the genetic associations found in large-scale studies is explicit, since most are not causal. Many of the associated variants are in LD with causal polymorphisms. They may act as eQTLs/sQTLs for other genes (as observed for *CELF1* rs10838725), located in regions of regulation of gene expression or ncRNA genes. The influence of lncRNAs on the regulation of genes, which can cause pathological disorders, is becoming increasingly evident (Cipolla et al., 2018; Salviano-Silva et al., 2018). Through the LD analysis performed for the four associated SNPs in our study, we were able to find six lncRNAs that are possibly playing a role in LOAD and which have not been analyzed until now. Some polymorphisms can lead to changes in the secondary structure of these lncRNAs, resulting in the loss or gain in the binding of miRNAs, probably deregulating essential pathways and, consequently, causing the disease. Experimental validation studies of these lncRNAs and their alleles in LOAD can contribute to a better understanding of the disease. Thus, our study brings new promising targets for future research on Alzheimer’s disease.

## Data Availability

The genotype data are available in the Supplementary Material and also at OSF via the link: https://osf.io/njau3/?view_only=949c6b1c91c94e33b2c3e4d152f82a0e.
