# Supplementary material for: GWAS-Top Polymorphisms Associated With Late-Onset Alzheimer Disease in Brazil: Pointing Out Possible New Culprits Among Non-Coding RNAs
Source: Front Mol Biosci. 2021 Jul 5;8:632314. doi: 10.3389/fmolb.2021.632314 (PMC8287568; doi:10.3389/fmolb.2021.632314)
Supplement: Supplementary file 1 [file DataSheet2.docx]

Supplementary Material

# Supplementary Table 1 - Databases used for *in silico* analysis.

| **DATABASE** | **ANALYZES PERFORMED IN THIS STUDY** | **URL** | **REFERENCE** |
| --- | --- | --- | --- |
| GWAS Catalog | Associated Diseases and traits | https://www.ebi.ac.uk/gwas/ | (Buniello et al., 2019) |
| DisGeNet |  | https://www.disgenet.org/home/ | (Piñero et al., 2017) |
| LDlink | Linkage disequilibrium | https://ldlink.nci.nih.gov/?tab=home | (Machiela and Chanock, 2015) |
| UCSC Genome Browser | Gene regulation | https://genome.ucsc.edu/cgi-bin/hgGateway | (Kent et al., 2002) |
| GTEx Portal | Gene expression and eQTLs / sQTLs search | https://www.gtexportal.org/home/ | (Ardlie et al., 2015) |
| Braineac |  | http://braineac.org/ | (Ramasamy et al., 2014) |
| Expression Atlas | Gene expression | https://www.ebi.ac.uk/gxa/home | (Papatheodorou et al., 2020) |
| Ensembl | General information about the gene / ncRNA | http://www.ensembl.org/index.html | (Yates et al., 2019) |
| GeneCard |  | https://www.genecards.org/ | (Fishilevich et al., 2017) |
| HGNC |  | https://www.genenames.org/ | (Bruford et al., 2020) |
| UniProt | Structure, expression, and function of the protein | https://www.uniprot.org/ | (Bateman et al., 2017) |
| Human Protein Atlas |  | https://www.proteinatlas.org/ | (Uhlén et al., 2015) |
| STRING | Protein Network | https://string-db.org/ | (Szklarczyk et al., 2019) |
| lncRNASNP2 | Search for SNPs in lncRNA region and the impact it on lncRNA secondary structure and miRNA interaction | http://bioinfo.life.hust.edu.cn/lncRNASNP/#!/ | (Miao et al., 2018) |
| miRNASNP v3 | Characteristics of miRNA | http://bioinfo.life.hust.edu.cn/miRNASNP/#!/ | (Gong et al., 2015) |
| NONCODE | Sequence, structure, and expression of ncRNA | http://www.noncode.org/ | (Fang et al., 2018) |
| LNCipedia | General information about the lncRNA and reports in the literature | https://lncipedia.org/ | (Volders et al., 2019) |
| LncBook |  | https://bigd.big.ac.cn/lncbook/index | (Ma et al., 2019) |
| RNACentral |  | https://rnacentral.org/ | (Sweeney et al., 2019) |
| TANRIC |  | https://cutt.ly/bd1HBRf | (Li et al., 2015) |
| Lncident | Identification of lncRNA | https://cutt.ly/1d1HMUP | (Han et al., 2016) |
| ncRPheno | Search for associations between ncRNAs and diseases | https://cutt.ly/Yd1H4ie | (Zhang et al., 2020) |
| LncRNADisease v2.0 |  | http://www.rnanut.net/lncrnadisease/ | (Bao et al., 2019) |
| LncBase v.2 | Prediction of lncRNA-miRNA interaction | https://cutt.ly/fd1HLb4 | (Paraskevopoulou et al., 2016) |
| ENCORI |  | http://starbase.sysu.edu.cn/index.php | (Li et al., 2014) |
| miRBase | General information about miRNAs | http://www.mirbase.org/index.shtml | (Kozomara et al., 2019) |
| TissueAtlas | miRNA expression in different tissues | https://ccb-web.cs.uni-saarland.de/tissueatlas/ | (Ludwig et al., 2016) |
| mirPath | Prediction of miRNA action pathways | http://snf-515788.vm.okeanos.grnet.gr/ | (Vlachos et al., 2015) |
| TarBase v.8 | Search for genes regulated by the miRNA found | https://cutt.ly/Ad1HCk7 | (Karagkouni et al., 2018) |
| RNAfold WebServer | Prediction of the lncRNA secondary structure | https://cutt.ly/Ud1H1G9 | (Lorenz et al., 2011) |

# Supplementary Table 2 – Results of logistic regression for all investigated SNPs.

| **REGION** | **SNP** |  | **O.R.** | **C.I. [95%]** | **P** | **Pc^#^** | **I. V.** | **HWE** | |
| --- | --- | --- | --- | --- | --- | --- | --- | --- | --- |
|  |  |  |  |  |  |  |  | **CON** | **PAT** |
| *ABCA7* | rs4147929 | *A/A* | 0.91 | 0.11-7.45 | 0.932 |  |  | 0.741 | 0.571 |
|  |  | *A/G* | 1.19 | 0.61-2.29 | 0.612 |  |  |  |  |
|  |  | *G/G* | 0.86 | 0.45-1.63 | 0.637 |  |  |  |  |
|  |  | *A+* | 1.17 | 0.61-2.23 | 0.637 |  |  |  |  |
|  |  | *G+* | 1.09 | 0.13-8.94 | 0.932 |  |  |  |  |
|  |  | additive model | 1.13 | 0.63-2.02 | 0.687 |  |  |  |  |
| *ADAMTS9-AS2* | rs704454 | *C/C* | 3.21 | 0.67-15.34 | 0.144 |  |  | 0.297 | 1 |
|  |  | *C/T* | 0.85 | 0.44-1.63 | 0.629 |  |  |  |  |
|  |  | *T/T* | 0.98 | 0.51-1.86 | 0.953 |  |  |  |  |
|  |  | *C+* | 1.02 | 0.54-1.94 | 0.953 |  |  |  |  |
|  |  | *T+* | 0.26 | 0.05-1.35 | 0.110 |  |  |  |  |
|  |  | additive model | 1.32 | 0.70-2.47 | 0.385 |  |  |  |  |
| *APOE* | rs769449 | *A/A** | - | - | - |  |  | 1 | 1 |
|  |  | *A/G* | 0.84 | 0.32-2.25 | 0.736 |  |  |  |  |
|  |  | ***G/G*** | **0.28** | **0.14-0.57** | **<0.000** | **0.0002** | **BMI** |  |  |
|  |  | ***A+*** | **3.55** | **1.76-7.19** | **<0.000** | **0.0002** | **BMI** |  |  |
|  |  | *G+** | - | - | - |  |  |  |  |
|  |  | **additive model** | **3.49** | **1.80-6.76** | **<0.000** | **0.0002** | **BMI** |  |  |
| *BIN1-CYP27C1* | rs6733839 | *C/C* | 0.55 | 0.29-1.05 | 0.070 |  | BMI, *ε4* | 0.281 | 0.865 |
|  |  | *C/T* | 1.30 | 0.70-2.41 | 0.406 |  |  |  |  |
|  |  | *T/T* | 1.96 | 0.76-5.05 | 0.165 |  |  |  |  |
|  |  | *C+* | 0.51 | 0.20-1.32 | 0.165 |  |  |  |  |
|  |  | *T+* | 1.81 | 0.95-3.43 | 0.070 |  | BMI, *ε4* |  |  |
|  |  | **additive model** | **1.62** | **1.01-2.59** | **0.045** | **0.049** | **BMI, *ε4*** |  |  |
|  | rs744373 | *A/A* | 0.69 | 0.36-1.29 | 0.246 |  |  | 0.242 | 0.856 |
|  |  | *A/G* | 0.98 | 0.51-1.86 | 0.948 |  |  |  |  |
|  |  | ***G/G*** | **2.83** | **1.04-7.66** | **0.041** | **0.049** | ***ε4*** |  |  |
|  |  | *A+* | 0.32 | 0.10-1.0 | 0.051 |  | BMI, *ε4* |  |  |
|  |  | *G+* | 1.45 | 0.77-2.74 | 0.246 |  |  |  |  |
|  |  | additive model | 1.55 | 0.96-2.51 | 0.076 |  | BMI, *ε4* |  |  |
| *CD2AP* | rs10948363 | *A/A* | 1.03 | 0.56-1.89 | 0.934 |  |  | 0.323 | 0.833 |
|  |  | *A/G* | 0.85 | 0.46-1.58 | 0.604 |  |  |  |  |
|  |  | *G/G* | 2.18 | 0.48-9.83 | 0.310 |  |  |  |  |
|  |  | *A+* | 0.46 | 0.10-2.06 | 0.310 |  |  |  |  |
|  |  | *G+* | 0.97 | 0.53-1.80 | 0.934 |  |  |  |  |
|  |  | additive model | 1.08 | 0.64-1.83 | 0.766 |  |  |  |  |

| **REGION** | **SNP** |  | **O.R.** | **C.I. [95%]** | | **P** | | **Pc^#^** | | **I.V.** | | **HWE** | | |
| --- | --- | --- | --- | --- | --- | --- | --- | --- | --- | --- | --- | --- | --- | --- |
|  |  |  |  |  |  |  |  |  |  |  |  | **CON** | | **PAT** |
| *CD33* | rs3865444 | *A/A* | 1.04 | | 0.34-3.17 | | 0.950 | |  | |  | 0.524 | 0.063 | |
|  |  | *A/C* | 1.12 | | 0.60-2.07 | | 0.723 | |  | |  |  |  |  |
|  |  | *C/C* | 0.88 | | 0.48-1.64 | | 0.695 | |  | |  |  |  |  |
|  |  | *A+* | 1.13 | | 0.61-2.10 | | 0.695 | |  | |  |  |  |  |
|  |  | *C+* | 0.96 | | 0.31-2.95 | | 0.950 | |  | |  |  |  |  |
|  |  | additive model | 1.09 | | 0.67-1.77 | | 0.736 | |  | |  |  |  |  |
| *CELF1* | rs10838725 | ***C/C*** | **0.21** | **0.04-0.99** | | **0.049** | | **0.049** | | **BMI, *ε4*** | | 0.660 | | 0.110 |
|  |  | *C/T* | 1.48 | 0.80-2.75 | | 0.212 | |  | |  | |  |  |  |
|  |  | *T/T* | 0.95 | 0.52-1.75 | | 0.875 | |  | |  | |  |  |  |
|  |  | *C+* | 1.05 | 0.57-1.93 | | 0.875 | |  | |  | |  |  |  |
|  |  | ***T+*** | **4.88** | **1.01-23.60** | | **0.049** | | **0.049** | | **BMI, *ε4*** | |  |  |  |
|  |  | additive model | 0.83 | 0.50-1.36 | | 0.453 | |  | |  | |  |  |  |
| *CLU* | rs11136000 | *C/C* | 1.01 | 0.52-1.95 | | 1.950 | |  | |  | | 0.121 | | 0.129 |
|  |  | *C/T* | 0.92 | 0.50-1.70 | | 0.800 | |  | |  | |  |  |  |
|  |  | *T/T* | 1.15 | 0.48-2.77 | | 0.750 | |  | |  | |  |  |  |
|  |  | *C+* | 0.87 | 0.36-2.08 | | 0.750 | |  | |  | |  |  |  |
|  |  | *T+* | 0.99 | 0.51-1.90 | | 0.974 | |  | |  | |  |  |  |
|  |  | additive model | 1.03 | 0.65-1.65 | | 0.883 | |  | |  | |  |  |  |
| *CTNNA2* | rs2974151 | *C/C* | 1.21 | 0.59-2.47 | | 0.601 | |  | |  | | 1 | | 1 |
|  |  | *C/G* | 0.79 | 0.38-1.63 | | 0.521 | |  | |  | |  |  |  |
|  |  | *G/G* | 1.67 | 0.12-23.27 | | 0.703 | |  | |  | |  |  |  |
|  |  | *C+* | 0.60 | 0.04-8.35 | | 0.703 | |  | |  | |  |  |  |
|  |  | *G+* | 0.83 | 0.40-1.69 | | 0.601 | |  | |  | |  |  |  |
|  |  | additive model | 0.88 | 0.46-1.69 | | 0.703 | |  | |  | |  |  |  |
| *EPHA1* | rs11771145 | *A/A* | 1.00 | 0.41-2.49 | | 0.992 | |  | |  | | 0.687 | | 0.065 |
|  |  | *A/G* | 0.68 | 0.37-1.26 | | 0.218 | |  | |  | |  |  |  |
|  |  | *G/G* | 1.48 | 0.79-2.74 | | 0.217 | |  | |  | |  |  |  |
|  |  | *A+* | 0.68 | 0.36-1.26 | | 0.217 | |  | |  | |  |  |  |
|  |  | *G+* | 0.99 | 0.40-2.46 | | 0.992 | |  | |  | |  |  |  |
|  |  | additive model | 0.82 | 0.52-1.28 | | 0.375 | |  | |  | |  |  |  |
| *INPP5D* | rs35349669 | *C/C* | 1.74 | 0.96-3.15 | | 0.065 | |  | | BMI | | 0.843 | | 1 |
|  |  | *C/T* | 0.68 | 0.37-1.25 | | 0.210 | |  | |  | |  |  |  |
|  |  | *T/T* | 0.76 | 0.31-1.87 | | 0.556 | |  | |  | |  |  |  |
|  |  | *C+* | 1.31 | 0.53-3.20 | | 0.556 | |  | |  | |  |  |  |
|  |  | *T+* | 0.58 | 0.31-1.09 | | 0.091 | |  | |  | |  |  |  |
|  |  | additive model | 0.70 | 0.45-1.11 | | 0.131 | |  | |  | |  |  |  |
| *MS4A6A* | rs610932 | *G/G* | 1.01 | 0.53-1.93 | | 0.966 | |  | |  | | 1 | | 1 |
|  |  | *G/T* | 0.87 | 0.47-1.61 | | 0.650 | |  | |  | |  |  |  |
|  |  | *T/T* | 1.28 | 0.55-2.99 | | 0.573 | |  | |  | |  |  |  |
|  |  | *G+* | 0.78 | 0.33-1.83 | | 0.573 | |  | |  | |  |  |  |
|  |  | *T+* | 0.99 | 0.52-1.87 | | 0.966 | |  | |  | |  |  |  |
|  |  | additive model | 1.06 | 0.68-1.66 | | 0.790 | |  | |  | |  |  |  |

| **REGION** | | **SNP** |  | | **O.R.** | | **C.I. [95%]** | | **P** | **Pc^#^** | **I.V.** | **HWE** | | | | |
| --- | --- | --- | --- | --- | --- | --- | --- | --- | --- | --- | --- | --- | --- | --- | --- | --- |
|  |  |  |  |  |  |  |  |  |  |  |  | **CON** | | | **PAT** | |
| *PICALM* | rs3851179 | | *C/C* | | 1.37 | | 0.73-2.55 | 0.319 | |  |  | | 0.671 | | | 0.855 |
|  |  |  | *C/T* | | 0.74 | | 0.40-1.38 | 0.351 | |  |  | |  |  |  |  |
|  |  |  | *T/T* | | 0.95 | | 0.35-2.60 | 0.918 | |  |  | |  |  |  |  |
|  |  |  | *C+* | | 1.05 | | 0.38-2.89 | 0.918 | |  |  | |  |  |  |  |
|  |  |  | *T+* | | 0.73 | | 0.39-1.35 | 0.319 | |  |  | |  |  |  |  |
|  |  |  | additive model | | 0.82 | | 0.52-1.32 | 0.423 | |  |  | |  |  |  |  |
| *ZCWPW1* | | rs1476679 | *C/C* | 0.71 | | 0.16-3.08 | | 0.644 | |  |  | | | 0.568 | | 1 |
|  |  |  | *C/T* | 1.31 | | 0.69-2.51 | | 0.408 | |  |  | | |  |  |  |
|  |  |  | *T/T* | 0.82 | | 0.44-1.54 | | 0.544 | |  |  | | |  |  |  |
|  |  |  | *C+* | 1.21 | | 0.65-2.27 | | 0.544 | |  |  | | |  |  |  |
|  |  |  | *T+* | 1.41 | | 0.32-6.17 | | 0.644 | |  |  | | |  |  |  |
|  |  |  | additive model | 1.10 | | 0.65-1.85 | | 0.732 | |  |  | | |  |  |  |
| *PTK2B* | | rs28834970 | *C/C* | 1.35 | | 0.56-3.28 | | 0.507 | |  |  | 0.837 | | | | 1 |
|  |  |  | *C/T* | 0.84 | | 0.46-1.56 | | 0.588 | |  |  |  |  |  |  |  |
|  |  |  | *T/T* | 1.03 | | 0.55-1.91 | | 0.935 | |  |  |  |  |  |  |  |
|  |  |  | *C+* | 0.97 | | 0.52-1.81 | | 0.935 | |  |  |  |  |  |  |  |
|  |  |  | *T+* | 0.74 | | 0.30-1.80 | | 0.507 | |  |  |  |  |  |  |  |
|  |  |  | additive model | 1.06 | | 0.68-1.66 | | 0.783 | |  |  |  |  |  |  |  |
| *SLC24A4* | | rs10498633 | *G/G* | 0.91 | | 0.47-1.76 | | 0.781 | |  |  | 0.320 | | | | 0.166 |
|  |  |  | *G/T* | 1.23 | | 0.62-2.43 | | 0.550 | |  |  |  |  |  |  |  |
|  |  |  | *T/T* | 0.46 | | 0.07-3.05 | | 0.421 | |  |  |  |  |  |  |  |
|  |  |  | *G+* | 2.17 | | 0.33-14.40 | | 0.421 | |  |  |  |  |  |  |  |
|  |  |  | *T+* | 1.10 | | 0.57-2.13 | | 0.781 | |  |  |  |  |  |  |  |
|  |  |  | additive model | 0.99 | | 0.56-1.76 | | 0.984 | |  |  |  |  |  |  |  |
| *SNCA* | | rs3857059 | *A/A* | 1.59 | | 0.75-3.37 | | 0.227 | |  |  | 0.236 | | | | 0.138 |
|  |  |  | *A/G* | 0.46 | | 0.21-1.04 | | 0.061 | |  | Schooling,  BMI,  AD in  family |  |  |  |  |  |
|  |  |  | *G/G* | 1.95 | | 0.20-18.96 | | 0.563 | |  |  |  |  |  |  |  |
|  |  |  | *A+* | 0.51 | | 0.05-4.96 | | 0.563 | |  |  |  |  |  |  |  |
|  |  |  | *G+* | 0.67 | | 0.30-1.47 | | 0.318 | |  |  |  |  |  |  |  |
|  |  |  | additive model | 0.91 | | 0.51-1.62 | | 0.740 | |  |  |  |  |  |  |  |

The values result from multivariate logistic regression (performed with STATA 9.2). In bold: significant P-value; underlined: trend; OR - *odds ratio*; CI - confidence interval; P – P-value; Pc# - P-value correct for false discovery rate; I.V. – independent variable; HWE – Hardy-Weinberg equilibrium; PAT – Patients; CON- Controls; BMI - Body mass index; * - not possible to calculate since all the controls presented the *rs769449*G* allele. *ABCA7 - ATP binding cassette subfamily A member 7*; *ADAMTS9-AS2 - ADAMTS9 antisense RNA 2*; *APOE- Apolipoprotein E*; *BIN1- Bridging integrator 1*; *CYP27C1 - Cytochrome P450 family 27 subfamily C member 1*; *CD2AP - CD2 associated protein*; *CD33 - CD33 molecule*; *CELF1 - CUGBP Elav-like family member 1*; *CLU – Clusterin*; *CTNNA2 - Catenin alpha 2*; *EPHA1 - EPH receptor A1*; *INPP5D - Inositol polyphosphate-5-phosphatase D*; *MS4A6A - Membrane spanning 4-domains A6A*; *PICALM - Phosphatidylinositol binding clathrin assembly protein*; *ZCWPW1 - Zinc finger CW-type and PWWP domain containing 1*; *PTK2B - Protein tyrosine kinase 2 beta*; *SLC24A4 - Solute carrier family 24 member 4*; *SNCA - Synuclein alpha*.

# Supplementary Table 3 – Linkage disequilibrium analysis.

| **rs10838725 – *CELF1* (*T*/*C*)** | | | | | | | | | | | | | | |
| --- | --- | --- | --- | --- | --- | --- | --- | --- | --- | --- | --- | --- | --- | --- |
| **ID** | **REGION** | **ALLELES** | **POSITION** | **CEU** | | | **TSI** | | | **IBS** | | | **CORRELATED ALLELES** | **eQTL/ sQTL^#^** |
|  |  |  |  | **MAF (%)** | **D’** | **r^2^** | **MAF (%)** | **D’** | **r^2^** | **MAF (%)** | **D’** | **r^2^** |  |  |
| rs10838725 | *CELF1* | T/C | Intron | 34 | Ref. | Ref. | 34 | Ref. | Ref. | 29 | Ref. | Ref. | Ref. | X |
| rs10838726 | *CELF1* | C/G | Intron | 34 | 1 | 1 | 34 | 1 | 1 | 29 | 1 | 1 | T=C,C=G | X |
| rs11039290 | *CELF1* | G/A | Intron | 34 | 1 | 1 | 34 | 1 | 1 | 29 | 1 | 1 | T=G,C=A | X |
| rs71475921 | *CELF1* | G/A | Intron | 34 | 1 | 1 | 34 | 1 | 1 | 29 | 1 | 1 | T=G,C=A | X |
| rs59288626 | *CELF1* | CT/- | Intron | 34 | 1 | 1 | 33 | 1 | 0.979 | 29 | 1 | 1 | T=CT,C= - | X |
| rs11039297 | *CELF1* | G/A | Intron | 34 | 1 | 1 | 34 | 1 | 1 | 29 | 1 | 1 | T=G,C=A | X |
| rs7933019 | *CELF1* | G/C | Intron | 34 | 1 | 1 | 34 | 1 | 0.979 | 29 | 1 | 1 | T=G,C=C | X |
| rs10838731 | *NDUFS3 - FAM180B* | C/T | Intergenic | 34 | 1 | 1 | 34 | 1 | 1 | 29 | 1 | 1 | T=C,C=T | X |
| rs71457224* | *C1QTNF4 - MTCH2* | -/T | Intergenic | 34 | 1 | 1 | 34 | 1 | 1 | 29 | 1 | 1 | T=-,C=T | No |
| rs7120548 | *MTCH2* | T/C | Intron | 34 | 1 | 1 | 34 | 1 | 1 | 29 | 1 | 1 | T=T,C=C | X |
| rs6485758 | *CELF1* | G/A | Intron | 34 | 1 | 0.978 | 34 | 1 | 0.979 | 29 | 1 | 1 | T=G,C=A | X |
| rs35188627 | *CELF1* | A/- | Intron | 33 | 1 | 0.978 | 34 | 1 | 1 | 29 | 1 | 0.977 | T=A,C= - | No |
| rs10769282* | *C1QTNF4 - MTCH2* | T/G | Intergenic | 32 | 1 | 0.912 | 32 | 1 | 0.939 | 28 | 1 | 0.955 | T=T,C=G | No |
| rs11039332 | *AGBL2* | G/A | Intron | 33 | 0.954 | 0.890 | 33 | 0.978 | 0.918 | 29 | 1 | 0.977 | T=G,C=A | X |
| rs12365079 | *AGBL2* | A/T | Intron | 33 | 0.954 | 0.890 | 33 | 0.978 | 0.918 | 29 | 1 | 0.977 | T=A,C=T | X |
| rs147311748 | *AGBL2* | G/T | Intron | 33 | 0.954 | 0.890 | 33 | 0.978 | 0.918 | 29 | 1 | 0.977 | T=G,C=T | X |
| rs12807014 | *FNBP4* | T/C | Intron | 33 | 0.954 | 0.890 | 32 | 0.978 | 0.898 | 28 | 1 | 0.955 | T=T,C=C | X |
| rs12577383 | *FNBP4* | C/T | Intron | 33 | 0.954 | 0.890 | 32 | 0.978 | 0.898 | 28 | 1 | 0.955 | T=C,C=T | X |
| rs7927445 | *FNBP4* | G/T | Intron | 33 | 0.954 | 0.890 | 32 | 0.978 | 0.898 | 28 | 1 | 0.955 | T=G,C=T | X |
| rs12223593 | *FNBP4* | T/G | Regulatory region | 33 | 0.954 | 0.890 | 32 | 0.978 | 0.898 | 28 | 1 | 0.955 | T=T,C=G | X |
| rs7114011 | *NUP160* | A/C | Intron | 33 | 0.954 | 0.890 | 32 | 0.978 | 0.898 | 28 | 1 | 0.955 | T=A,C=C | X |
| rs11039398 | *NUP160* | C/T | Intron | 33 | 0.954 | 0.890 | 32 | 0.978 | 0.898 | 28 | 1 | 0.955 | T=C,C=T | X |
| rs7934481 | *NUP160 - PTPRJ* | C/T | Intergenic | 33 | 0.954 | 0.890 | 32 | 0.956 | 0.858 | 29 | 0.977 | 0.933 | T=C,C=T | X |
| rs12287076 | *NDUFS3 - FAM180B* | G/C | Intergenic | 31 | 1 | 0.871 | 32 | 1 | 0.939 | 26 | - | - | T=C,C=G | X |
| rs35333514 | *FNBP4* | -/AAAC | Intron | 33 | 0.953 | 0.869 | 34 | 0.916 | 0.821 | 28 | 1 | 0.955 | T= -,C=AAAC | No |
| rs10458914 | *NUP160* | G/A | Intron | 33 | 0.953 | 0.869 | 32 | 0.978 | 0.898 | 28 | 1 | 0.955 | T=G,C=A | X |
| rs7131262 | *NUP160* | T/A | Intron | 33 | 0.953 | 0.869 | 32 | 0.978 | 0.898 | 28 | 1 | 0.955 | T=T,C=A | X |
| rs61895112 | *CELF1* | T/C | Intron | 30 | 1 | 0.850 | 33 | 1 | 0.979 | 27 | 1 | 0.890 | T=T,C=C | X |
| rs147850410 | *CELF1* | G/A | Intron | 30 | 1 | 0.850 | 33 | 1 | 0.979 | 27 | 1 | 0.890 | T=G,C=A | No |
| rs11039283 | *CELF1* | G/A | Intron | 30 | 1 | 0.850 | 33 | 1 | 0.979 | 27 | 1 | 0.890 | T=G,C=A | No |
| rs11039284 | *CELF1* | A/G | Intron | 30 | 1 | 0.850 | 33 | 1 | 0.979 | 27 | 1 | 0.890 | T=A,C=G | X |
| rs12225051 | *CELF1* | C/T | Intron | 30 | 1 | 0.850 | 33 | 1 | 0.979 | 27 | 1 | 0.890 | T=C,C=T | X |
| rs2280231 | *NDUFS3* | C/T | 5'UTR | 30 | 1 | 0.850 | 33 | 1 | 0.979 | 27 | 1 | 0.890 | T=C,C=T | X |
| rs7118178 | *MTCH2* | G/A | Intron | 30 | 1 | 0.850 | 33 | 1 | 0.979 | 27 | 1 | 0.890 | T=G,C=A | X |
| rs11039244 | *RAPSN* | G/A | Intron | 38 | 1 | 0.839 | 35 | 0.979 | 0.90 | 32 | 1 | 0.876 | T=G,C=A | X |
| rs2293576 | *SLC39A13* | G/A | Exon | 38 | 1 | 0.839 | 35 | 0.979 | 0.900 | 31 | 0.977 | 0.853 | T=G,C=A | X |
| rs12224672 | *CELF1* | C/T | Intron | 30 | 1 | 0.830 | 33 | 1 | 0.979 | 27 | 1 | 0.890 | T=C,C=T | X |
| rs12361415 | *RAPSN-CELF1* | T/G | Intergenic | 31 | 0.975 | 0.828 | 35 | 0.979 | 0.919 | 27 | 1 | 0.890 | T=T,C=G | X |
| rs6485795 | *NUP160-PTPRJ* | G/A | Intergenic | 32 | 0.952 | 0.827 | 33 | 0.957 | 0.878 | 29 | 0.977 | 0.933 | T=G,C=A | X |
| rs12577643 | *RAPSN* | A/T | Intron | 37 | 0.976 | 0.817 | 32 | 0.956 | 0.858 | 32 | 1 | 0.876 | T=A,C=T | X |
| rs11039266 | *CELF1* | T/G | Intron | 30 | 0.975 | 0.808 | 34 | 0.979 | 0.939 | 27 | 1 | 0.890 | T=T,C=G | X |
| rs11039406 | *NUP160* | C/T | Intron | 31 | 0.951 | 0.807 | 33 | X | X | 28 | X | X | T=C,C=T | No |
| rs71457231 | *FNBP4-NUP160* | -/T | Intergenic | 32 | 0.929 | 0.806 | 32 | 0.956 | 0.858 | 28 | 0.953 | 0.868 | T= -,C=T | No |
| rs7103835 | *RAPSN* | G/A | Intron | 34 | x | x | 35 | 0.957 | 0.880 | 29 | X | X | T=G,C=A | X |
| rs68010938 | *SLC39A13* | A/- | Intron | 34 | x | x | 35 | 0.957 | 0.880 | 29 | X | X | T=A,C= - | No |
| rs11039364 | *FNBP4* | C/T | Intron | 30 | x | x | 32 | 0.978 | 0.878 | 26 | 1 | 0.869 | T=C,C=T | X |
| rs10838703 | *SLC39A13* | C/G | Intron | 35 | x | x | 35 | 0.957 | 0.861 | 29 | X | X | T=C,C=G | X |
| rs12803857 | *NUP160-PTPRJ* | G/A | Intergenic | 30 | x | x | 32 | 0.956 | 0.839 | 27 | 0.975 | 0.847 | T=G,C=A | X |
| rs10838791 | *NUP160-PTPRJ* | C/T | Intergenic | 27 | x | x | 32 | 0.956 | 0.839 | 28 | 0.928 | 0.804 | T=C,C=T | X |
| rs1017730 | *AGBL2* | G/A | Intron | 27 | x | x | 29 | X | X | 25 | 1 | 0.807 | T=G,C=A | X |
| **rs769449 – *APOE* (*G*/*A*)** | | | | | | | | | | | | | | |
| rs769449 | *APOE* | G/A | Intron | 15 | Ref. | Ref. | 9 | Ref. | Ref. | 8 | Ref. | Ref. | Ref. | X |
| rs10414043* | *AC011481.4* | G/A | Non-coding | 14 | 1 | 1 | 10 | 1 | 0.895 | 8 | 1 | 1 | G=G,A=A | X |
| rs7256200* | *AC011481.4* | G/T | Non-coding | 14 | 1 | 0.960 | 10 | 1 | 0.895 | 8 | 1 | 1 | G=G,A=T | No |
| rs429358* | *APOE* | T/C | Exon | 18 | x | x | 10 | 1 | 0.850 | 14 | x | x | G=T,A=C | X |
| **rs6733839 – *BIN1-CYP27C1* (*C*/*T*)** | | | | | | | | | | | | | | |
| rs6733839 | *BIN1-CYP27C1* | *C/T* | Intergenic | 40 | Ref. | Ref. | 42 | Ref. | Ref. | 31 | Ref. | Ref. | Ref. | No |
| rs4663105* | *BIN1-CYP27C1* | *A/C* | Intergenic | 43 | 1 | 0.865 | 43 | 1 | 0.944 | 36 | 1 | 0.828 | C=A,T=C | X |
| **rs744373 – *BIN1-CYP27C1* (*A*/*G*)** | | | | | | | | | | | | | | |
| rs744373* | *BIN1-CYP27C1* | A/G | Intergenic | 30 | Ref. | Ref. | 29 | Ref. | Ref. | 25 | Ref. | Ref. | Ref. | No |
| rs730482* | *BIN1-CYP27C1* | A/T | Intergenic | 30 | 1 | 1 | 29 | 1 | 1 | 25 | 1 | 1 | A=A, G=T | No |

The variants marked with (*) are present in the long noncoding RNA (lncRNA) region; # - variant that has an expression and or splicing quantitative trait locus (eQTL / sQTL) effect on the brain or whole blood; Populations of: CEU - Utah with northern and western European ancestry; TSI – Toscana; IBS – Iberian; MAF – minor allele frequence; *CELF1 - CUGBP Elav-like family member 1*; *NDUFS3* – *NADH - ubiquinone oxidoreductase core subunit S3*; *FAM180B* - *Family with sequence similarity 180 member B*; *C1QTNF4 –* *C1q and TNF related 4*; *MTCH2 – Mitochondrial carrier 2*; *AGBL2 – ATP/GTP binding protein like 2*; *FNBP4 – Formin binding protein 4*; *NUP160* - *Nucleoporin* 160; PTPRJ *– Protein tyrosine phosphatase receptor type J*; *SLC39A13 – Solute carrier family 39 member 13*; *APOE- Apolipoprotein E*; *BIN1- Bridging integrator* 1; CYP27C1 *- Cytochrome P450 family 27 subfamily C member 1*.

# Supplementary Table 4 – Genotype data

The genotype data are available in an excel spreadsheet due to the amount of data.

# Supplementary Figure 1 – Variants in linkage disequilibrium with rs10838725.


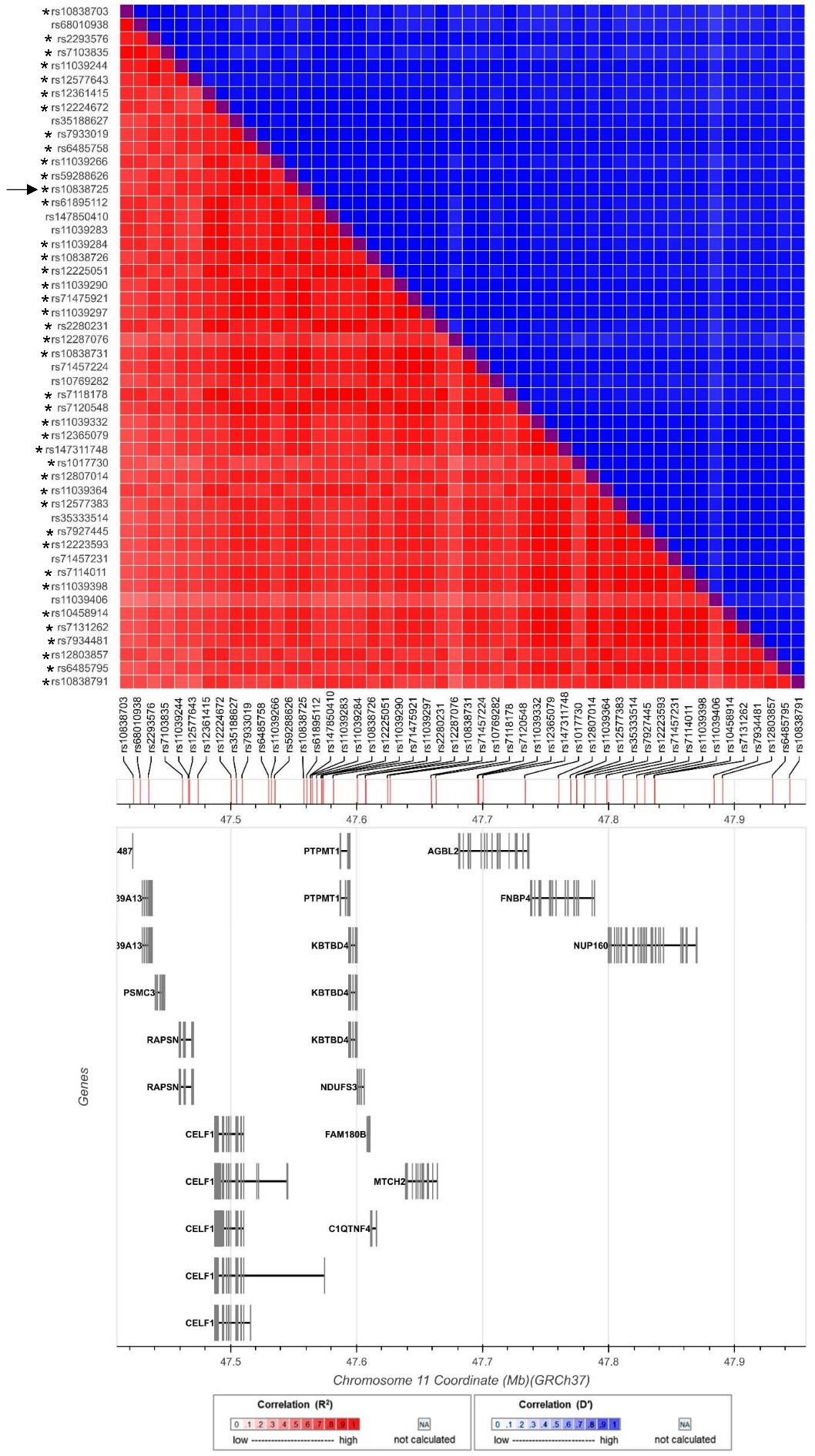


The variants marked with (*) have an expression and or splicing quantitative trait locus (eQTL / sQTL) effect on the brain or whole blood. The LD block was created based in three populations: CEU - Utah with northern and western European ancestry; TSI – Toscana; IBS – Iberian.

# Supplementary Figure 2 – Variants in linkage disequilibrium with rs769449.


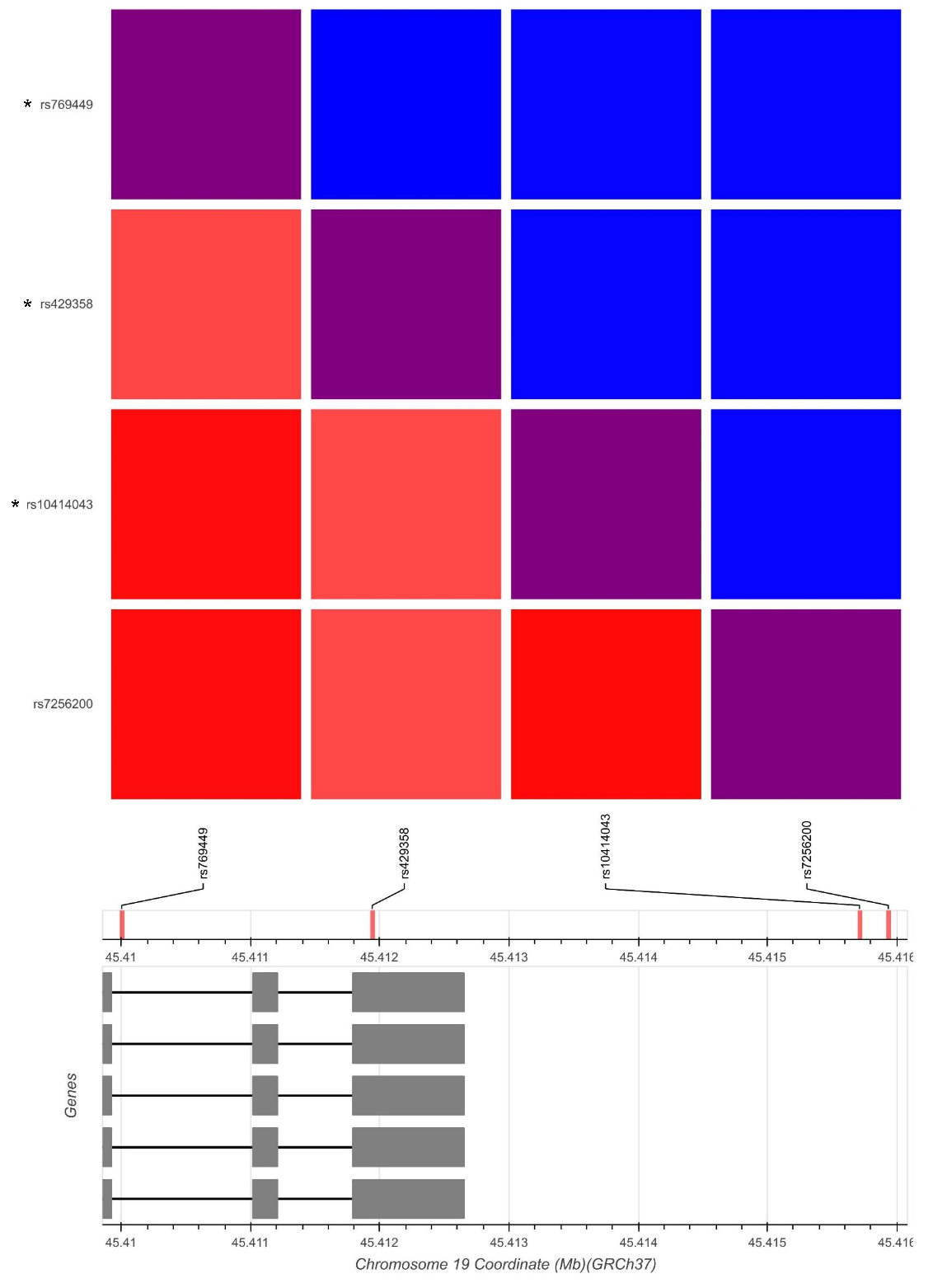

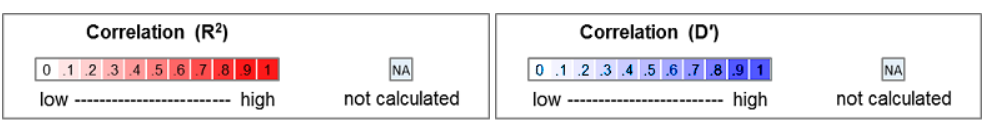


The variants marked with (*) have an expression and or splicing quantitative trait locus (eQTL / sQTL) effect on the brain or whole blood. The LD block was created based in three populations: CEU - Utah with northern and western European ancestry; TSI – Toscana; IBS – Iberian.

# Supplementary Figure 3 – Network of lncRNA associations with diseases.


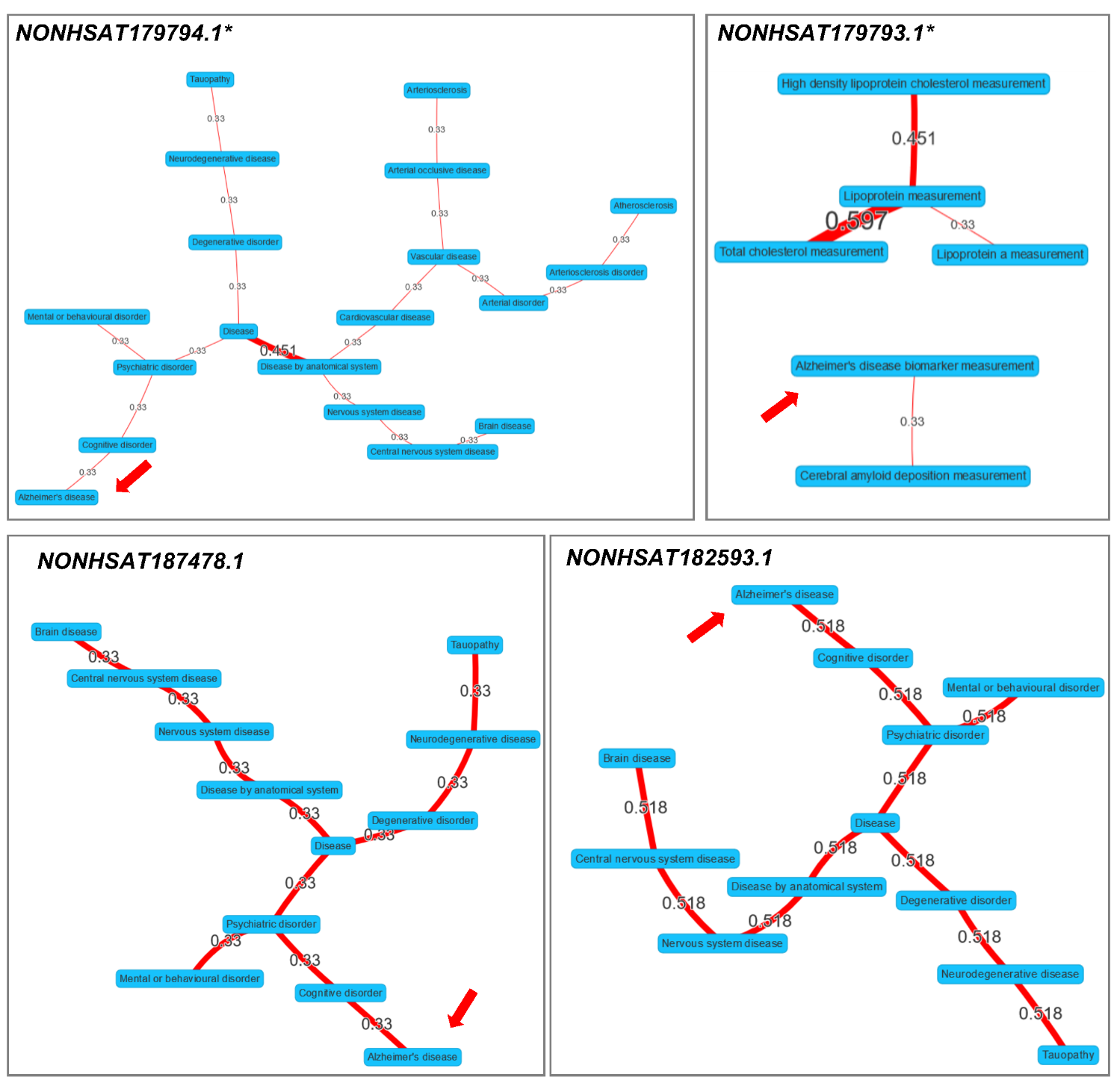


* These networks are not shown in full because of their size (they focus on brain diseases). These networks were generated through ncRPheno, a comprehensive database that provides experimentally supported associations between noncoding RNAs and disease phenotypes.
